# Killing two birds with one stone: How the respiratory syncytial virus polymerase initiates transcription and replication

**DOI:** 10.1371/journal.ppat.1007548

**Published:** 2019-02-28

**Authors:** Sarah L. Noton, Chadene Z. Tremaglio, Rachel Fearns

**Affiliations:** 1 Department of Microbiology, Boston University School of Medicine, Boston, Massachusetts, United States of America; 2 Department of Biology, University of Saint Joseph, West Hartford, Connecticut, United States of America; University of Kentucky, UNITED STATES

## Introduction

**Fig 1:**
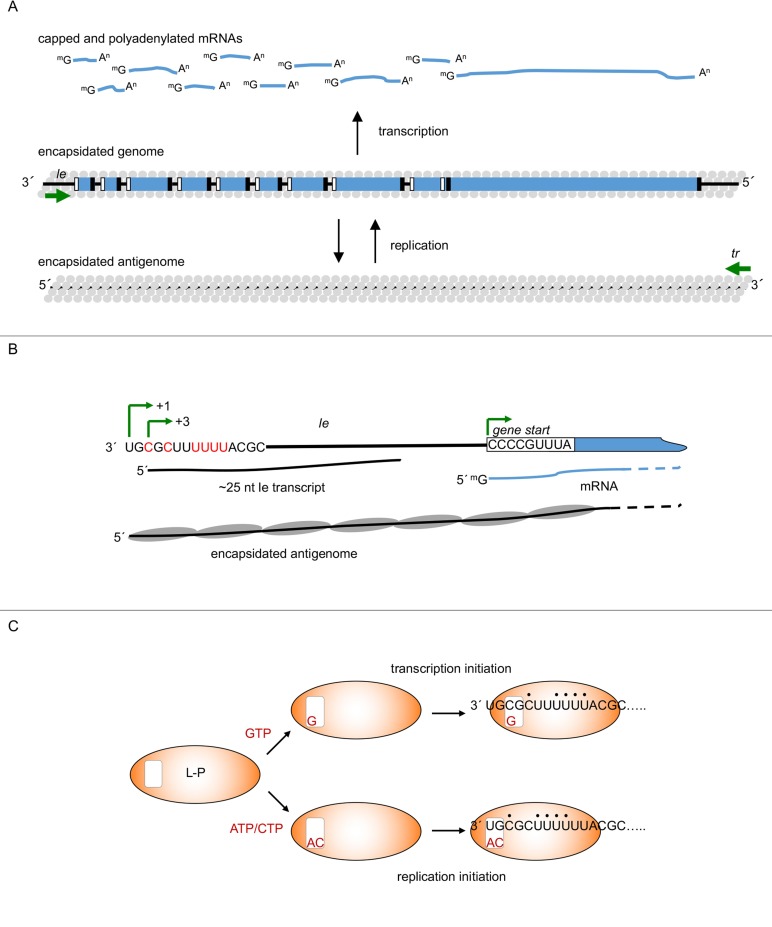
Schematic diagrams illustrating the mechanisms of RSV transcription and replication initiation. (A) Overview of the processes of transcription and replication, showing the capped and polyadenylated mRNAs and encapsidated antigenome and genome RNAs. The genes are shown as blue rectangles, with the g*ene start* and *gene end* signals represented by white and black boxes. The *le* and *tr* promoter regions are indicated with green arrows. The *le* promoter yields mRNAs containing a methylguanosine cap (^m^G) and polyadenylate tail (A^n^) and encapsidated antigenome; the *tr* promoter yields encapsidated genome RNA. The N protein that encapsidates the genome and antigenome RNA is shown as gray circles. Note that there is a gradient of transcription, which is not depicted here. (B) Initiation sites and RNAs produced from the 3ʹ end of the genome. The schematic shows the *le* region and the beginning of the first gene. The nucleotides in red are required for both transcription and replication, and are identical to the RSV L *gs* signal (CCCUGUUUUA). The *NS1* gene is shown in a blue partial rectangle, with its *gs* signal shown in a white box. The initiation sites are shown with green arrows: those at 3C and the first *gs* signal are necessary for transcription; the initiation site at 1U is required for replication. The N protein is represented as a gray oval. It seems likely that if there were insufficient N protein available for encapsidation, RNA initiated at 1U would also be released after approximately 25 nt, allowing the polymerase to engage in transcription. (C) Model for initiation at two sites on the promoter. The L-P complex is represented with an orange oval. The polymerization active site, containing the NTP_1_ and NTP_2_ binding sites, is shown as a white box. The L-P complex could bind in two different registers on the promoter, with stability for one position or the other being conferred by the bound GTP, or ATP/CTP. The black dots indicate nucleotides that are repeated in the promoter sequence that could allow binding in two registers. *le*, leader; RSV, respiratory syncytial virus; *tr*, trailer.

Respiratory syncytial virus (RSV) is the major cause of respiratory disease in infants and young children; it is also a significant problem in the elderly [1, 2]. RSV is a nonsegmented, negative strand RNA virus (nsNSV). Like other viruses in this group, the genome is a template for two distinct processes: transcription, which yields capped and polyadenylated mRNAs, and replication, which yields an encapsidated antigenome RNA. The antigenome, in turn, acts as a template for genome synthesis (Fig 1A; [3]). RSV encodes its own RNA-dependent RNA polymerase, which is responsible for both transcription and replication. This presents an intriguing puzzle, namely, how does the RSV polymerase perform both activities from the same template?

### What distinguishes transcription and replication?

Transcription and replication are both initiated from a promoter in the *leader* (*le)* region at the 3ʹ end of the genome [4–6]. During transcription, the polymerase is able to generate mRNAs by responding to *gene start* (*gs)* and *gene end* (*ge)* signals that flank each gene (Fig 1A) [7]. The *gs* signal directs the polymerase to initiate RNA synthesis. By analogy with related viruses, its complement at the 5ʹ end of the nascent RNA also has a function, directing the polymerase to add a methylated cap [8–10]. The *ge* signal directs the polymerase to polyadenylate and release the mRNA [7]. The polymerase can then scan the genome to locate the next *gs* signal and reinitiate RNA synthesis [11]. Some polymerase disengage from the template at each gene junction, resulting in a decreasing abundance of transcripts from the 3ʹ to the 5ʹ end of the genome [12]. This simple arrangement allows the viral genes to be expressed at appropriate levels relative to each other. During replication, the polymerase disregards the *ge* sequences as it moves along the genome, allowing it to proceed to the end of the genome to produce antigenome RNA. The *trailer* (*tr*) promoter at the 3ʹ end of the antigenome then signals genome synthesis (Fig 1A). The ability of the polymerase to override the *ge* signals as it is producing the antigenome is probably due to replicative RNA becoming encapsidated with nucleoprotein (N) as it is synthesized [13–15]. Thus, encapsidation is a key factor distinguishing transcription and replication.

### One promoter, two processes: How does the polymerase become engaged in either transcription or replication?

The answer lies in the fact that the promoter contains two initiation sites, one for each process. The first 11 nucleotides of the *le* are sufficient to signal initiation of RNA synthesis, and studies using a minigenome system showed that both transcription and replication depend on *le* nucleotides 3, 5, 8, 9, 10, and 11, a motif that bears strong similarity to a *gs* signal (although it is not identical) [6, 16, 17]. Primer extension analysis of *le* transcripts from RSV-infected cells showed that they were initiated either opposite the 3ʹ terminus of the genome at position 1U, or opposite the *gs*-like signal at 3C (Fig 1B; [17]). The RNA initiated at 1U can be encapsidated to produce antigenome. In contrast, RNA initiated at 3C is not efficiently encapsidated and is released after a short distance (approximately 25 nucleotides). The polymerase can then reinitiate RNA synthesis at the first *gs* signal to begin transcription [17, 18]. Synthesis of the short *le* RNA provides a mechanism by which the polymerase can break its association with the 3ʹ promoter and access the internal *gs* signal, which contains the sequence required for it to synthesize capped mRNA.

### Can the same polymerase initiate transcription and replication?

The core polymerase consists of the large polymerase subunit (L) and a cofactor, the phosphoprotein (P; [19, 20]. These proteins are sufficient for RNA polymerization, but other viral proteins, M2-1 and N protein, are required for transcription and replication, respectively [21, 22], raising the possibility that a different polymerase complex initiates at each site. For example, if an L-P-N complex were specifically able to initiate at 1U, this could explain why RNA initiated from this site is encapsidated, whereas RNA initiated from 3C is not. However, M2-1 is only required for transcription elongation and does not affect initiation [23], and in vitro studies showed that L-P complexes were capable of initiating at either 1U or 3C in the absence of M2-1 or N [20, 24, 25]. Therefore, the L-P complex alone can initiate transcription or replication and then mature into a fully competent transcriptase or replicase during elongation. Recruitment of N protein appears to depend on the sequence at the 5ʹ end of the nascent RNA, with the 5ʹ AC playing a key role [6, 15, 26]. This explains why RNA initiated at 1U becomes encapsidated and elongated, whereas RNA initiated at 3C is not.

### But how does the polymerase initiate from two different sites in the promoter?

The mechanism by which the RSV polymerase initiates at 1U or 3C was initially hinted at with minigenome assays. If position 1U of the promoter was substituted with a C residue or deleted, the polymerase was still able to perform RNA replication at approximately 60% of wild-type levels. The replication products were initiated at the wild-type position 1 with a nontemplated, wild-type ATP, such that templates containing a mutated *tr* promoter of sequences 3ʹ_GCUC… and 3ʹ GGCUC… yielded products of 5ʹ ACGAG…, a result that was confirmed using an in vitro assay [25, 27]. Likewise, minigenome studies in which the first C residue of a *gs* sequence was mutated showed the polymerase retained a strong preference for initiating mRNAs with GTP [28]. These studies indicate that the polymerase has an innate affinity for ATP and GTP, independently of the template nucleotides. There is evidence that the second nucleotide in the replication product (NTP_2_), CTP, also binds the polymerase independently of template sequence [25, 26]. In vitro studies revealed that varying ATP or GTP concentrations affected initiation at 1U or 3C. Increasing ATP augmented initiation from 1U and diminished initiation at 3C, whereas increasing GTP concentration had the opposite effect, indicating that these NTPs compete for the same binding site on the polymerase. Although the concentration of CTP had a strong effect on the efficiency of initiation from 1U, it did not affect initiation from 3C, suggesting that it binds a different site [25]. Together, these data suggest the model shown in Fig 1C. According to this model, the polymerase has two binding sites for NTP_1_ and NTP_2_. The NTP_1_ site has affinity for ATP or GTP, and CTP can occupy the NTP_2_ site. The polymerase then selects either the 1U or 3C initiation site depending on which NTPs it has bound. Inspection of the RSV promoter shows that nucleotides 3, 6, 7, 8, and 9, and 5, 8, 9, 10, and 11, each contain a 3ʹ-CXXUUUU motif. This repeating motif gives the potential for the polymerase to bind the template in two possible registers, such that its active site is opposite either positions 1 and 2 to initiate replication, or positions 3 and 4 to initiate transcription [25].

### How are the relative levels of mRNA, antigenome, and genome synthesis controlled?

**Fig 2:**
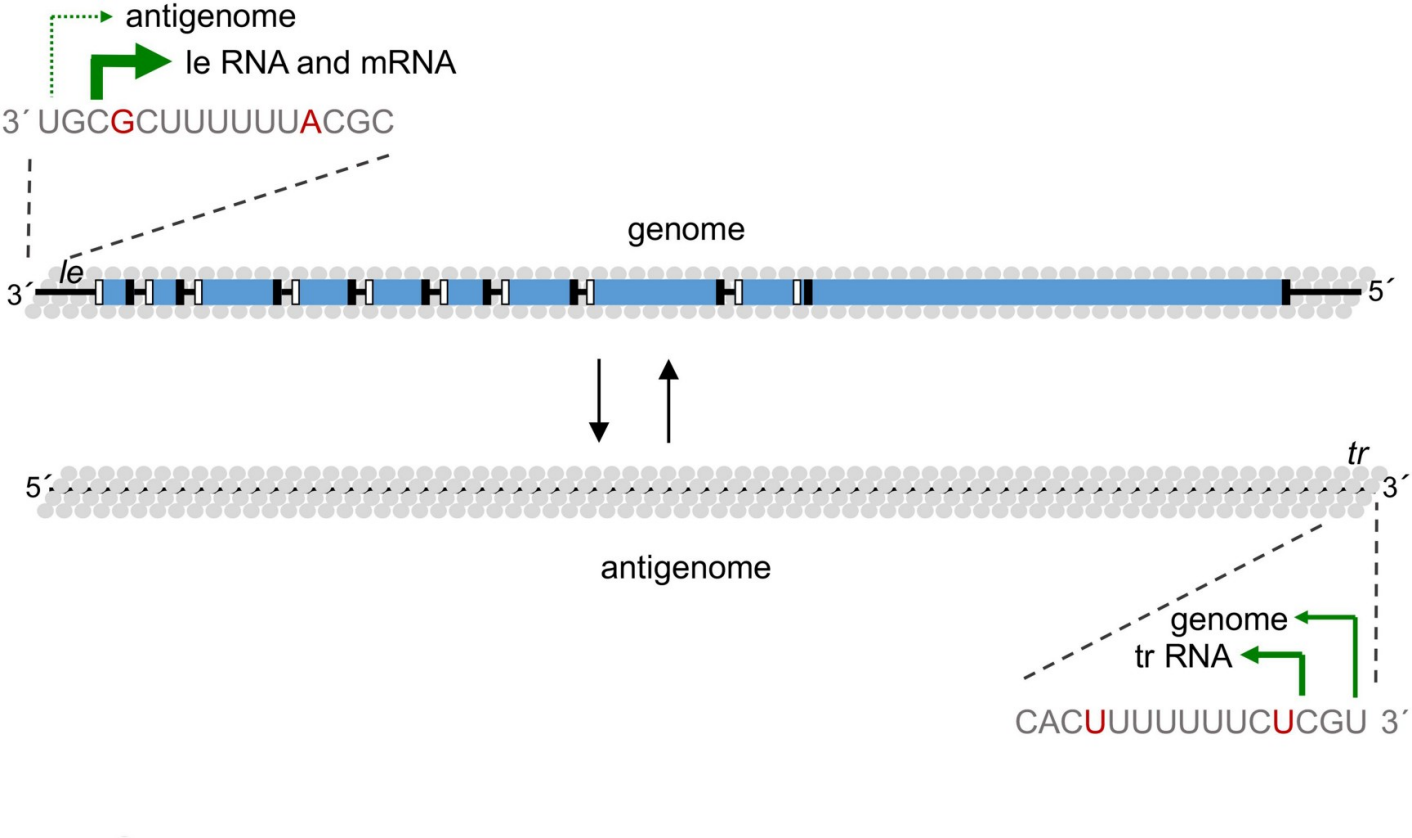
Schematic diagram illustrating the relative levels of initiation from positions 1 and 3 of the *le* and *tr* promoters. The genome and antigenome are shown as described in Fig 1A. The sequences of the *le* and *tr* promoters are shown in gray, with nucleotide differences shown in red. The green arrows show the initiation sites, with the weight of the arrows representing, approximately, the relative levels of initiation from each site. Note that the *tr* promoter also generates an approximately 25 nt RNA from position 3. The function of this RNA is not known, but it may be involved in subverting the cellular stress granule response [37]. *le*, leader; *tr*, trailer.

Transcription and replication products are present at different levels in RSV-infected cells, with the mRNAs being dominant, and genome RNA levels exceeding those of antigenome. Given that there is only one promoter in the *le* region, and the *le* and *tr* promoters are almost identical, this raises the question of how different amounts of RNAs are produced. Evidence indicates this is controlled by relative NTP concentrations and promoter sequence [25]. Like other polymerases, the RSV polymerase requires a high concentration of initiating NTPs (ATP and GTP), but only initiation at 1U requires a very high concentration of NTP_2_ (CTP). Because CTP is only present at low concentrations in cells [29], this would present a barrier to replication initiation. Additionally, the RSV polymerase had a propensity to initiate internally opposite 3 rather than opposite position 1, independently of its affinity for NTPs. These factors lead to transcription initiation being dominant compared to replication initiation at the *le* promoter. The relative levels of RNAs are also explained by small differences between the *le* and *tr* promoters. There are only two nucleotide differences within the first 12 nucleotides of the *le* and *tr*, at positions 4 and 12, and the *tr* promoter also directs RNA synthesis from 1U and 3C. However, studies comparing promoter activities showed that the *le* has a much greater bias towards initiation from position 3 versus position 1 than the *tr*, and mutation analysis linked this to the nucleotides at positions 4 and 12 [6, 25]. Therefore, these small differences between the two promoters contribute to a hierarchy of RNA production of mRNA, genome, and antigenome (Fig 2). A question that remains unanswered is how are transcription and replication regulated during the course of RSV infection? Transcriptases and replicases probably proceed along the genome with different kinetics due to the pausing that occurs at gene junctions during mRNA polyadenylation and capping. Therefore, it seems likely that mRNA and antigenome synthesis are regulated so that genome templates are dedicated to one process or the other. Although N protein is required to encapsidate the antigenome, because the transcriptase initiates at 3C, increasing N protein does not repress mRNA synthesis [30]. Instead, it is intriguing to speculate that temporal or spatial variations in NTP concentrations in infected cells govern the polymerase between transcription and replication, but this possibility has not yet been explored.

### Do other viruses use the same mechanism of transcription and replication initiation?

The nsNSVs, to which RSV belongs, are a large order of viruses. Most nsNSVs have a similar genome structure and use a similar strategy to transcribe and replicate it [31]. This raises the question of whether other nsNSVs use a similar mechanism as RSV to initiate transcription and replication. The answer to this is unclear, but data suggest that there are differences between families in the order. Human metapneumovirus (HMPV), which is in the same family as RSV (Pneumoviridae), shares a very similar promoter sequence, suggesting that it follows the same mechanism of transcription and replication initiation [3]. In contrast, in the case of the Paramyxoviridae, although transcription and replication both occur from a promoter at the 3ʹ end of the *le* region, similarly to RSV, there is no evidence for an internal initiation site, suggesting that both processes initiate from position 1. Consistent with this, in the paramyxoviruses, it appears that increasing N protein elicits a switch from transcription to replication [3, 32, 33]. In the case of the Rhabdoviridae, experiments using nucleocapsids isolated from virions indicate that both replication and transcription initiate at position 1 [34–36]. However, experiments analyzing transcription in cells, or using polymerase purified from cells, show that transcription initiates directly at the start of the first gene, suggesting that the 3ʹ end of the rhabdovirus nucleocapsid becomes reordered following cell entry, to expose the first *gs* signal [3, 34, 35]. Therefore, there seems to be considerable diversity between viruses in the order. However, despite this variability, many nsNSVs begin replication with ATP and CTP, suggesting that the ability of the polymerase to bind initiating NTPs independently of the template sequence might be a common hallmark of these viruses.

### Concluding remarks

In conclusion, RSV has evolved an elegant mechanism for initiating transcription and replication, which achieves several ends: First, it allows the viral polymerase to initiate both processes from a single promoter. Second, an innate affinity for ATP and CTP would lend the polymerase additional stability during initiation opposite the 3ʹ terminal nucleotide, a challenging event for viruses with linear genomes. And finally, the RSV promoter sequences have evolved so that different viral RNAs are produced in the appropriate amounts. Therefore, RSV provides an exquisite example of how a virus is able to accomplish multiple objectives with minimal genetic information.
